# Stoma-related complications and emergencies

**DOI:** 10.1186/s12245-022-00421-9

**Published:** 2022-05-09

**Authors:** Rodrick Babakhanlou, Kelly Larkin, Angel G. Hita, John Stroh, Sai-Ching Yeung

**Affiliations:** 1 Department of Leukemia, The University of Texas, MD Anderson Cancer Center, 515 Holcombe Blvd., Houston, TX 77030 USA; 2 Department of Emergency Medicine, The University of Texas, MD Anderson Cancer Center, 1515 Holcombe Blvd., Houston, TX 77030 USA

**Keywords:** Stoma, Ostomy, Stoma complications, Ileostomy, Colostomy, Urostomy, Parastomal herniation, Stoma necrosis, Stoma obstruction, High-output stoma, Stoma care

## Abstract

Stoma creations are common procedures in surgical specialties. They can be created either as a temporary or a permanent measure. Despite advancements in surgical technique and stoma care, complications are common. Patients experiencing stoma-related complications often present to the emergency department. Emergency physicians are not expected to be stoma experts, yet they are often the first point of contact for patients experiencing stoma-related complications. Accordingly, emergency physicians should be familiar with the types of stomas and complications and emergencies associated with them so that they can appropriately address the problems related to stomas. This article will provide a review of emergencies and complications associated with ileostomies, colostomies, and urostomies.

## Introduction

The term “stoma” comes from the Greek word meaning “mouth” and is interchangeably used with “ostomy.” A stoma is a surgical opening between a hollow organ and the body surface that is created when an anastomosis is not possible, either due to high risk of failure or when there is nothing distally to attach to [1].

Stomas can be created in the gastrointestinal tract (colostomies and ileostomies) and in the urogenital tract (urostomies) as either temporary or permanent solutions for redirecting stool or urine content. Stoma creation can be done in either an elective surgery or emergency setting [2]. The United Ostomy Association estimates that slightly more than 500,000 Americans now have some type of stoma [3]. In the USA, roughly 150,000 people undergo a creation of either a colostomy or an ileostomy annually [3, 4].

Nonetheless, complications are prevalent, despite advancements in the formation and care of ostomies [5]. Emergency physicians are not expected to be stoma experts, yet they are often the first point of contact for patients experiencing stoma-related complications. Accordingly, emergency physicians should be familiar with the types of stomas and the complications and emergencies associated with them, so that they can appropriately address them. This article will provide a review of emergencies and complications associated with ileostomies, colostomies, and urostomies.

## Main text

### Ileostomy and colostomy

Ostomies are commonly formed as diverting measures in the management of inflammatory bowel diseases, gastrointestinal malignancies, and bowel obstructions, perforations, and trauma [1, 6].

Temporary stoma may be created during emergency surgeries, such as in trauma or hollow viscus perforations, or as part of a planned, multi-step surgical procedure as a protective measure, where they serve as protective measures to prevent fecal content from reaching a distal bowel segment and causing anastomotic complications [1, 7]. Permanent ostomies are formed when the anorectum has been removed, such as in cancer patients or those suffering from inflammatory bowel diseases, or in cases where an anastomosis is not possible, such as in trauma- or radiation-related complications [7].

The most common indications for the creation of ostomies are outlined in Table 1.

**Table 1:** Indications for an ostomy

Cancer and related complications (obstruction)	
Complications related to diverticular disease	
Complications related to inflammatory bowel diseases (Crohn’s, ulcerative colitis)	
Trauma	
Bowel perforation	
Radiation enteritis	
Protection of distal anastomosis	
Treatment of anastomotic leak	

Outlined are indications for the formation of ostomies

Ileostomies are preferably created in the right abdomen through the rectus muscle. There are two types of ileostomies: loop ileostomy and end ileostomy [1]. During the creation of a loop ileostomy, a loop of the small intestine is pulled out through an abdominal incision and sutured to the skin. This type of stoma has two openings, which connect to the ascending and descending segment of the bowel [1].

An end ileostomy is created after total proctocolectomy and the descending bowel segment is pulled out through the abdominal incision and sutured to the skin.

**Fig. 1:**
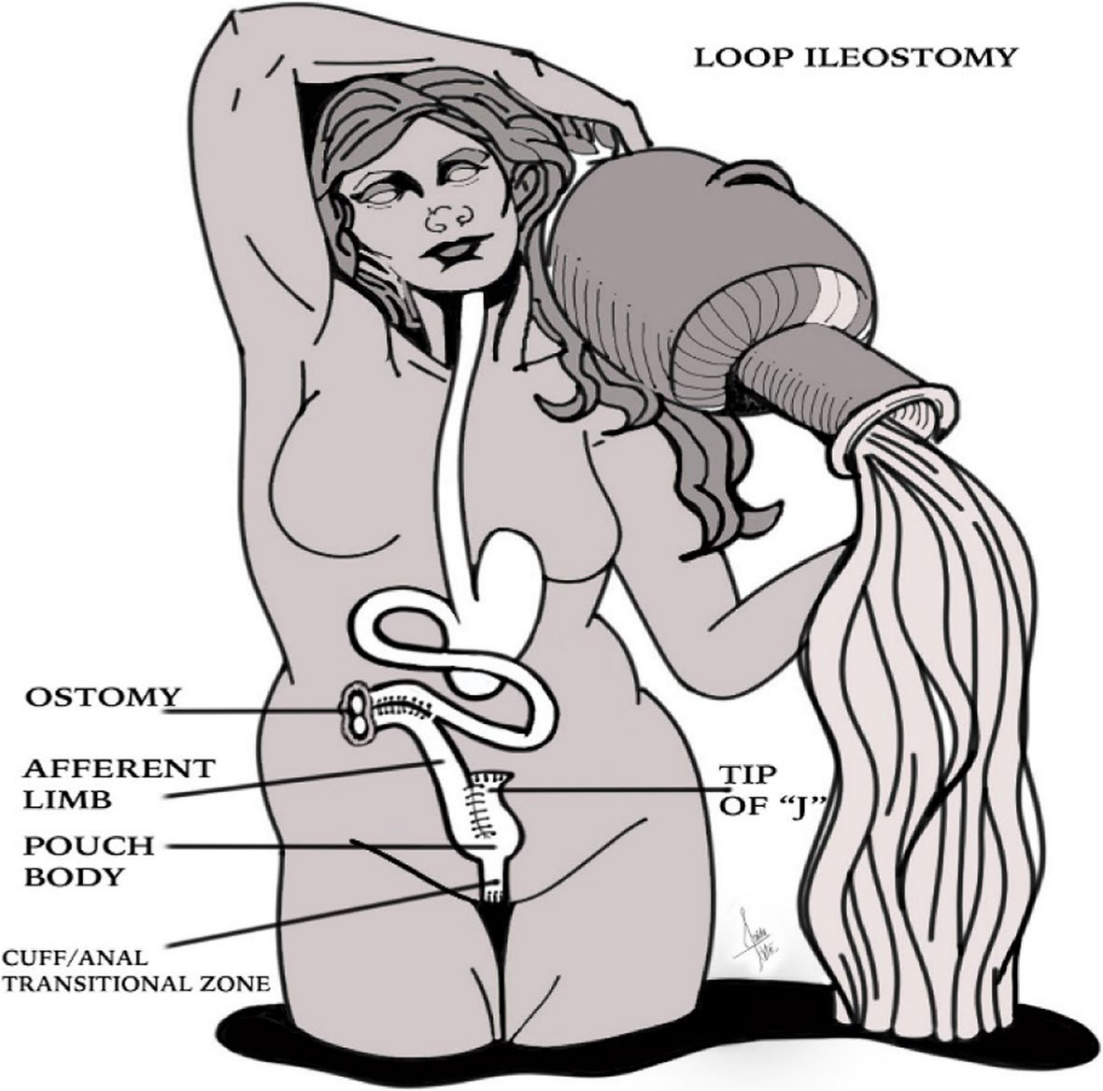
Loop ileostomy. A loop of the small intestine is pulled out through an abdominal incision and sutured to the skin. This type of stoma has two openings, which connect to the ascending and descending segment of the bowel

**Fig. 2:**
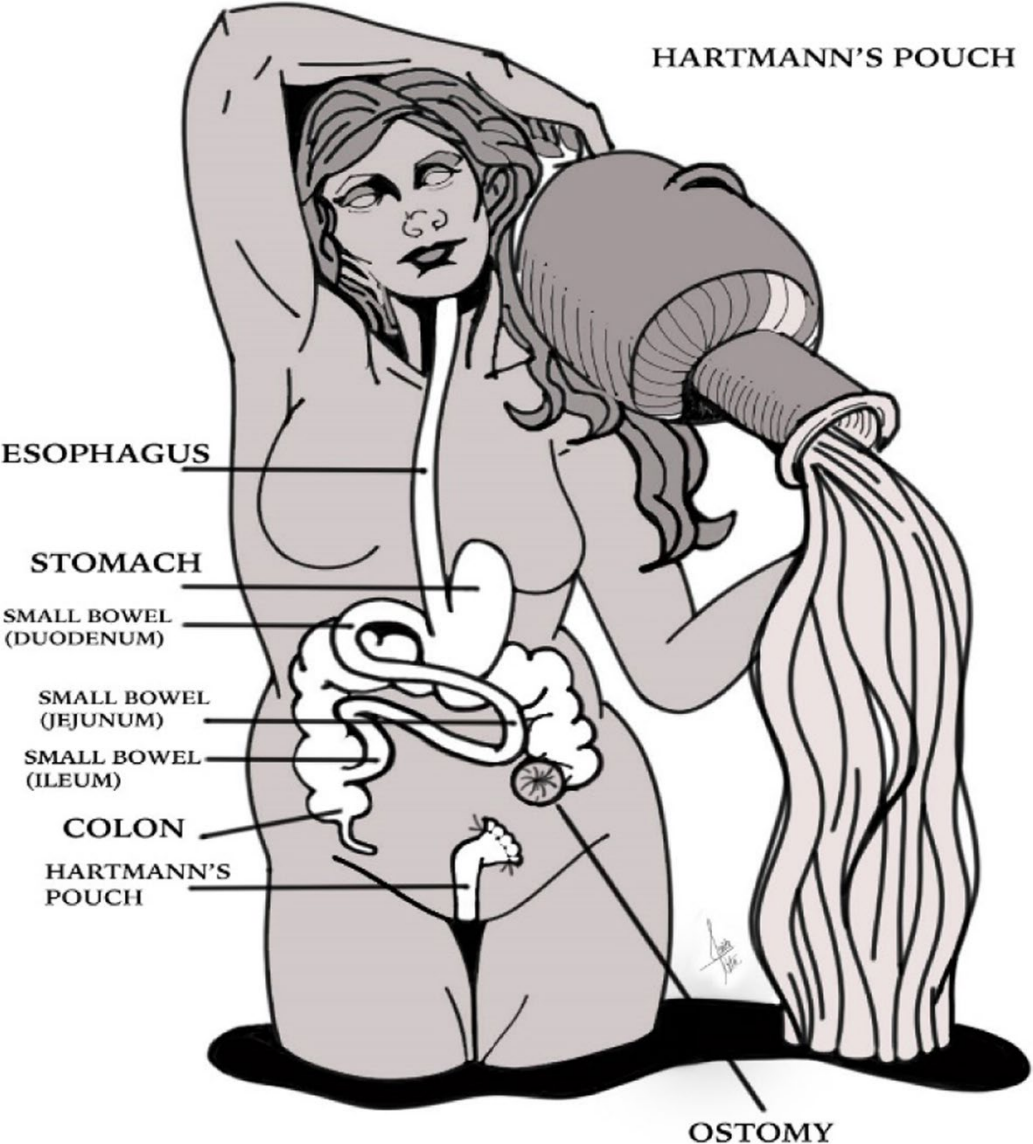
Hartmann pouch. Colostomies are preferably placed in the left abdomen. There are two types of colostomies: end colostomies and loop end colostomies. In end colostomies, a rectal stump is being left behind, known as Hartmann’s pouch

Colostomies are preferably placed in the left abdomen. As with ileostomy, there are two types of colostomies: end colostomies and loop end colostomies [8] (Figs. 1 and 2).

Stoma-related complications are common and can be classified as early or late. The rates of stoma complications reported in the literature vary widely, ranging from 10 to 70% [7, 9, 10]. The risk for complications from stoma creation is lifelong, although complications have been reported to be most frequent in the first 5 years postoperatively [7, 9]. Common early complications include leakage and skin irritations, high output resulting in fluid and electrolyte imbalances, or stoma necrosis; late complications include parastomal hernia, stoma prolapse, and stoma stenosis [7]. These can develop as a result of surgical- or patient-related factors (Table 2) [2, 10–12].

**Table 2:** Risk factors associated with stoma complications

Patient-related factors	Medical and surgical risk factors
Cardiac co-morbidities	Emergency surgery
Respiratory co-morbidities	Surgery for malignancy
Musculoskeletal co-morbidities	Poor surgical technique
Diabetes	Surgeon’s experience and specialty
Smoking	No preoperative input from a stoma nurse
Cancer	Concomitant chemotherapy
Obesity (BMI > 30)	Corticosteroid therapy
Age (> 60 years)	Preoperative radiation
Poor nutritional status	

Outlined are risk factors associated with stoma-related complications. These can be both patient-related, but also due to medical conditions that pose risk factors for the development of complications

### Early complications

#### Skin irritation

**Fig. 3:**
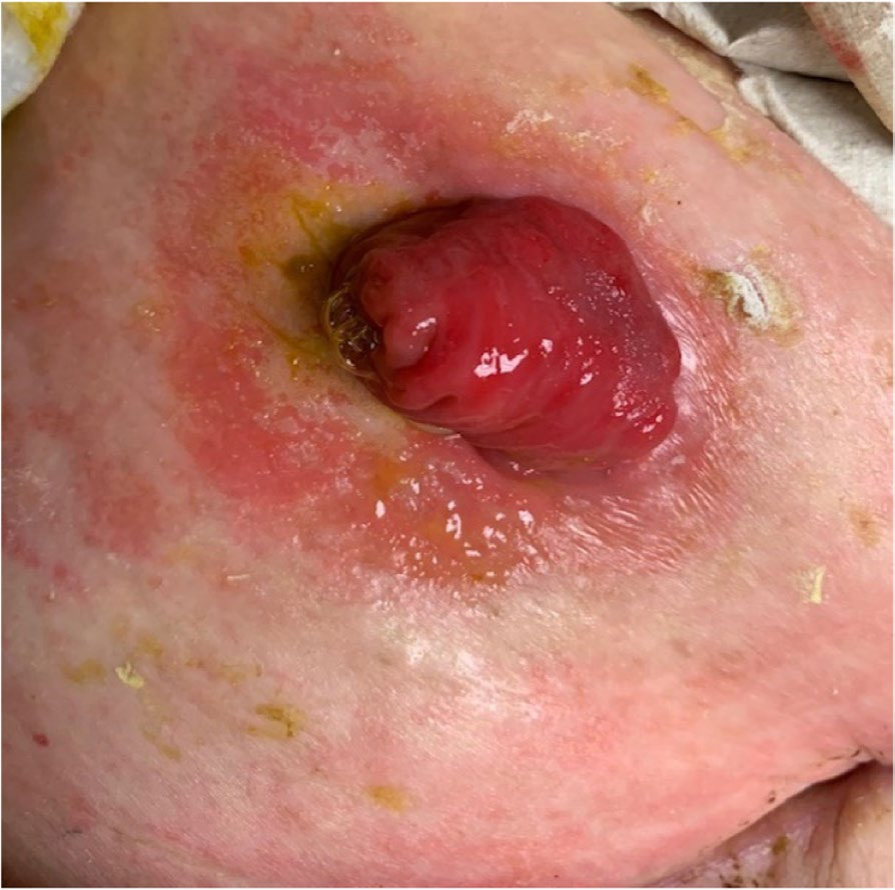
Peristomal skin irritation. Peristomal skin irritation is a commonly observed complication in all stoma types, which is related to the enzymatically active content of the output

Despite advancements in ostomy procedures as well as improvements with ostomy systems, patients continue to experience irritation of the skin surrounding the ostomy (Fig. 3). This is the most frequently observed complication with all stoma types [2, 7, 12]. Because the absorptive capacity of the colon is being bypassed, patients with an ileostomy have a watery output with a highly alkaline and active enzymatic content that can be extremely toxic and irritative to the skin [13]. Risk factors include obesity, diabetes and leakage due to a large aperture in the flange or a skin crease [2, 7, 13]. A variety of peristomal skin problems ranging from mild dermatitis to severe ulcerations can be encountered. Patients may present with itching and excoriations or a sore on the skin as a result of irritant contact dermatitis or even with bacterial or fungal infections [2]. In the emergency department, a visual inspection of the ostomy bag is important to ensure that it is properly fitted. If the skin is wet, erosive, and inflamed, early involvement of a stoma nurse is extremely important to ensure appropriate care and follow-up. Before a new stoma bag is applied, the skin should be cleaned and dried carefully. If bacterial or fungal infections are suspected, antifungal or antibacterial creams or powders should be applied [2]. If the skin is too wet, skin protectants can be used to facilitate absorption of fluid from the oozing skin and support the application of a new stoma bag. Skin protectants are available in form of powders, sprays, creams, or gels. They contain hydrocolloid that covers broken skin by creating a gel layer, which absorbs moisture and creates a dry coating, thus enabling adhesion of stoma appliance [14]. Other protectants act as a barrier against stoma effluent and skin maceration [14]. Occasionally barrier creams can cause adhesion problems with ostomy systems and caution is advised when removing those in the emergency department.

When fitting a new stoma bag, the aperture should be resized that it is not too large; otherwise, it may allow bowel content to come in contact with the skin worsening the skin irritation. Ideally, an ostomy appliance should not be changed more than once every 3–7 days in order to avoid skin breakdown [13]. However, should the patient report skin irritation or leakage, the appliance needs to be removed to reduce the risk of damage from feces or urine.

#### High-output stomas

A high-output ostomy is defined as a stoma with more than 1500 ml of daily output. High-output ostomies occur in 16% of new stoma creations and are more frequent in ileostomies than colostomies, given that the absorptive capacity of the large bowel is bypassed in an ileostomy [15]. Although the output of an ileostomy in the early postoperative period can reach more than 2000 ml, it does settle over several weeks to an average of 200–700 ml daily [16]. High-output ileostomies result from extensive small bowel resection or involvement of the small bowel in patients with Crohn’s disease, radiation enteritis, or infectious enteritis [16]. Patients are at high risk for dehydration, acute kidney injury, and electrolyte abnormalities, including hyponatremia, hypokalemia, and hypomagnesemia with resultant secondary complications [7, 16]. The risk of dehydration is very high during the early postoperative period, in patients suffering from chemotherapy-related nausea and diarrhea, and in those affected by infectious enteritis, as the oral intake of fluids is limited and insufficient to meet the demands. Various studies assessing the impact of dehydration on patients with ileostomies have found that hospital readmissions were primarily due to dehydration [17–19].

In the emergency department, an extensive workup should include blood tests to identify acute renal injury, but also include monitoring fluid intake and both urine and stoma output in order to calculate the fluid balance and to facilitate advanced care. Management of these patients should include parenteral hydration and correction of the electrolytes. Emergency physicians should have a low threshold for admitting these patients to the inpatient units, or they should at least observe and assess the patient for 24 h in the clinical decision unit, as improper management or even an early discharge may increase morbidity and mortality. The use of antidiarrheal medications, such as loperamide or tincture of opium can be used as adjuncts, as long as the patient’s primary care team is consulted beforehand.

#### Stoma necrosis

Ischemia is the most common cause of necrosis and is often related to tension on the mesentery, ligation of the primary blood vessel, or excessive mesenteric dissection [20]. Necrosis develops in up to 16% of patients, often in obese patients and those undergoing emergency stoma creation [7, 15]. Signs of ischemia arise within 24 h postoperatively. Clinically the stump of the stoma may have become discolored and patients may complain of a sore stoma site [15]. Although stoma necrosis is a rare complication, this condition is an emergency and requires urgent consultations with surgical services. While minor stoma discolorations may be carefully watched in the early postoperative period without the requirement of surgical intervention, severe necrosis requires timely revision of the stoma.

### Late complications

#### Parastomal hernia

Parastomal hernias are defined as incisional hernias associated with an abdominal wall stoma. They comprise the largest proportion of stoma-related complications requiring surgical intervention [5, 15]. Incidence rates vary by the type of stoma and range from 3 to 50% [2, 4, 15]. Risk factors for parastomal hernias include obesity, malnutrition, smoking, steroid use, chronic obstructive pulmonary disease, the presence of ascites, and advanced age [2, 15]. Parastomal hernias are usually asymptomatic. As their size increases, patients begin experiencing symptoms, such as discomfort, difficulty in maintaining an adequate appliance skin seal and the resulting skin irritation, and more seriously, obstruction, strangulation, or even perforation [4, 5, 15]. Although life-threatening complications are rare, impaired stoma output, severe pain or signs of necrosis, and shock should prompt the emergency physician to consult surgical services for immediate assessment.

#### Stomal prolapse

A prolapse happens when a proximal segment of bowel intussuscepts and protrudes through the stomal orifice [5]. A stoma prolapse occurs in 3% of ileostomies and in up to 10% of colostomies. Transverse loop colostomies are extremely susceptible to prolapse with an incidence rate of 30% [4, 7, 15]. Risk factors for the development of a stomal prolapse include obesity, conditions associated with increased abdominal pressure, or a poor surgical technique [4, 7]. The main symptom is skin irritation, difficulty fitting appliances, ulcerations, and bleeding, which can be managed conservatively in the outpatient setting [7, 15, 20]. Emergencies are rare but include ischemia and strangulation, which warrant prompt surgical review.

#### Stomal stenosis and obstruction

Stenosis is reported in 2–15% of stomas and can develop at any time during the postoperative period [4, 7]. Risk factors for the development of strictures and stenosis include ischemia, necrosis, retraction, or fistula formation. These features are frequently observed in patients with Crohn’s disease [4, 7]. The most common clinical sign is a noisy flatus. A stenosis rarely represents an emergency, but can lead to an obstruction, especially caused by food particles, when not chewed properly. Signs and symptoms of an obstruction include nausea, vomiting, and a thin clear liquid output with a foul odor or no output at all, in association with abdominal distension, pain, and cramping. To confirm a stomal obstruction, a physician can check for a local blockage by inserting a finger into the stoma. A computed tomography with oral contrast can also be helpful to locate the obstruction [21]. If an obstruction is found, an urgent surgical consult is warranted. When patients are not able to tolerate oral intake, the placement of a nasogastric tube and administration of intravenous fluids are warranted followed by admission to surgical services.

### Urostomy

**Fig. 4:**
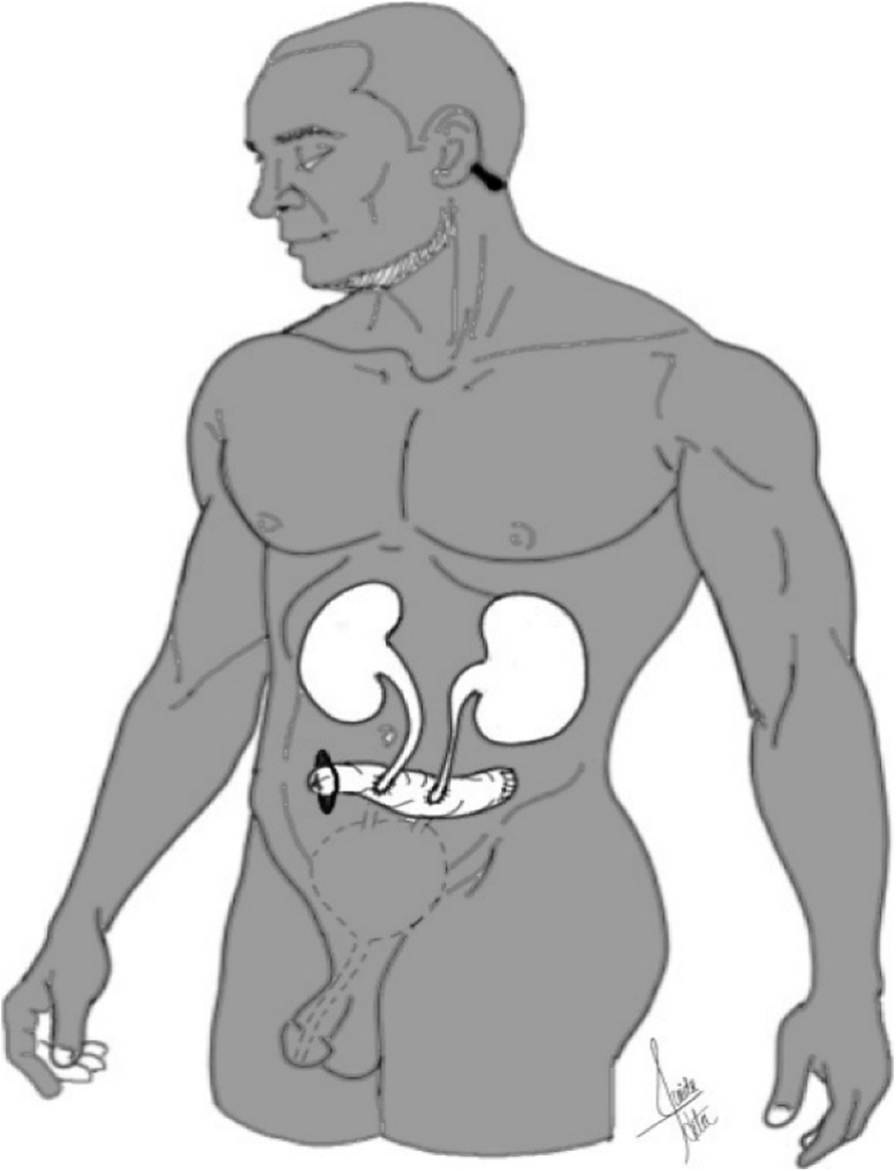
Non-continent cutaneous urinary diversion. Urine is drained from the ureters to a conduit, constructed from the ileum or the colon, and anastomosed to the abdominal skin surface, where the urine is collected into an external appliance

**Fig. 5:**
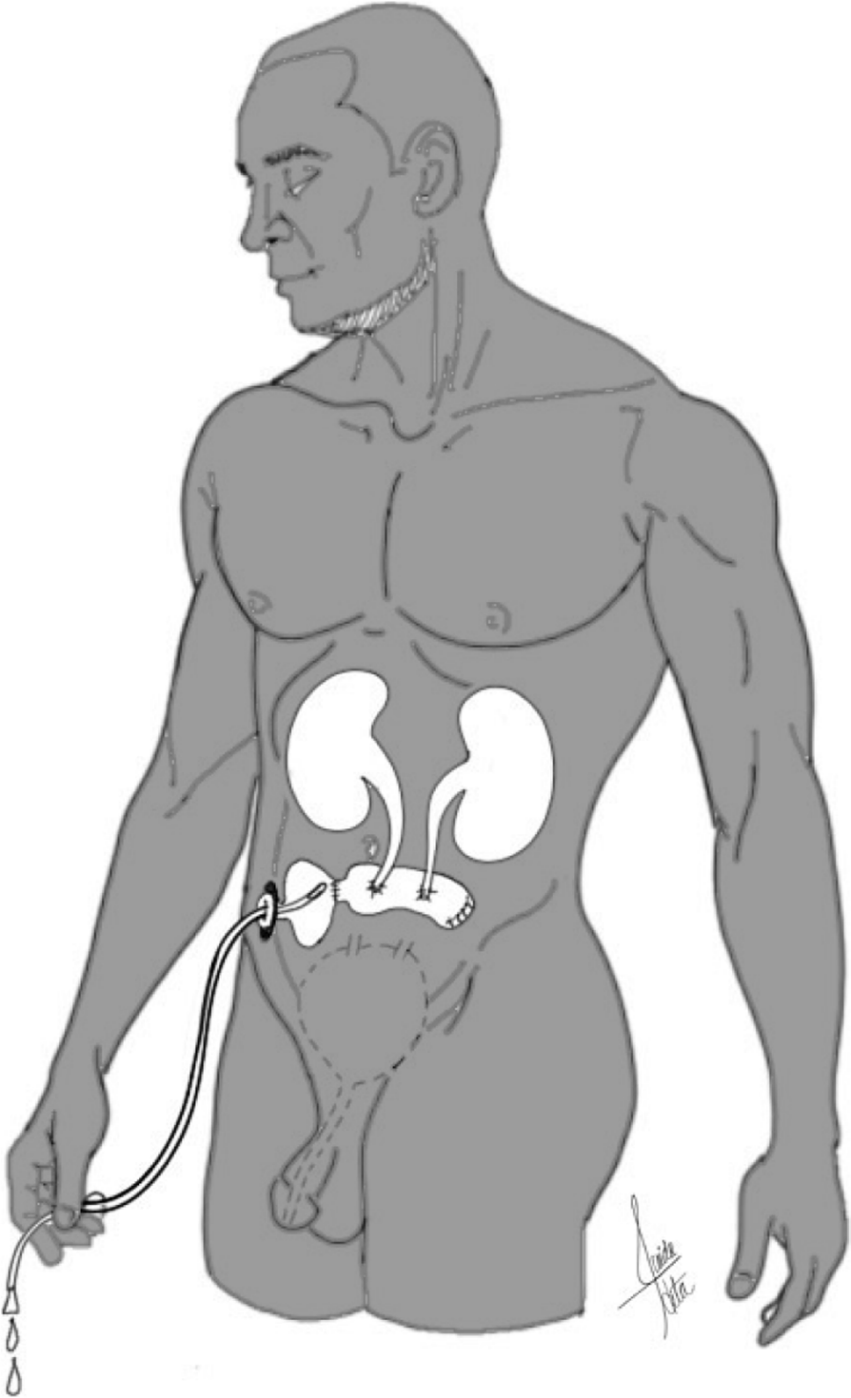
Continent cutaneous urinary diversion. The ureters are attached to a urinary pouch, created from a bowel segment (ileum or colon), which then is brought to the skin as a stoma

**Fig. 6:**
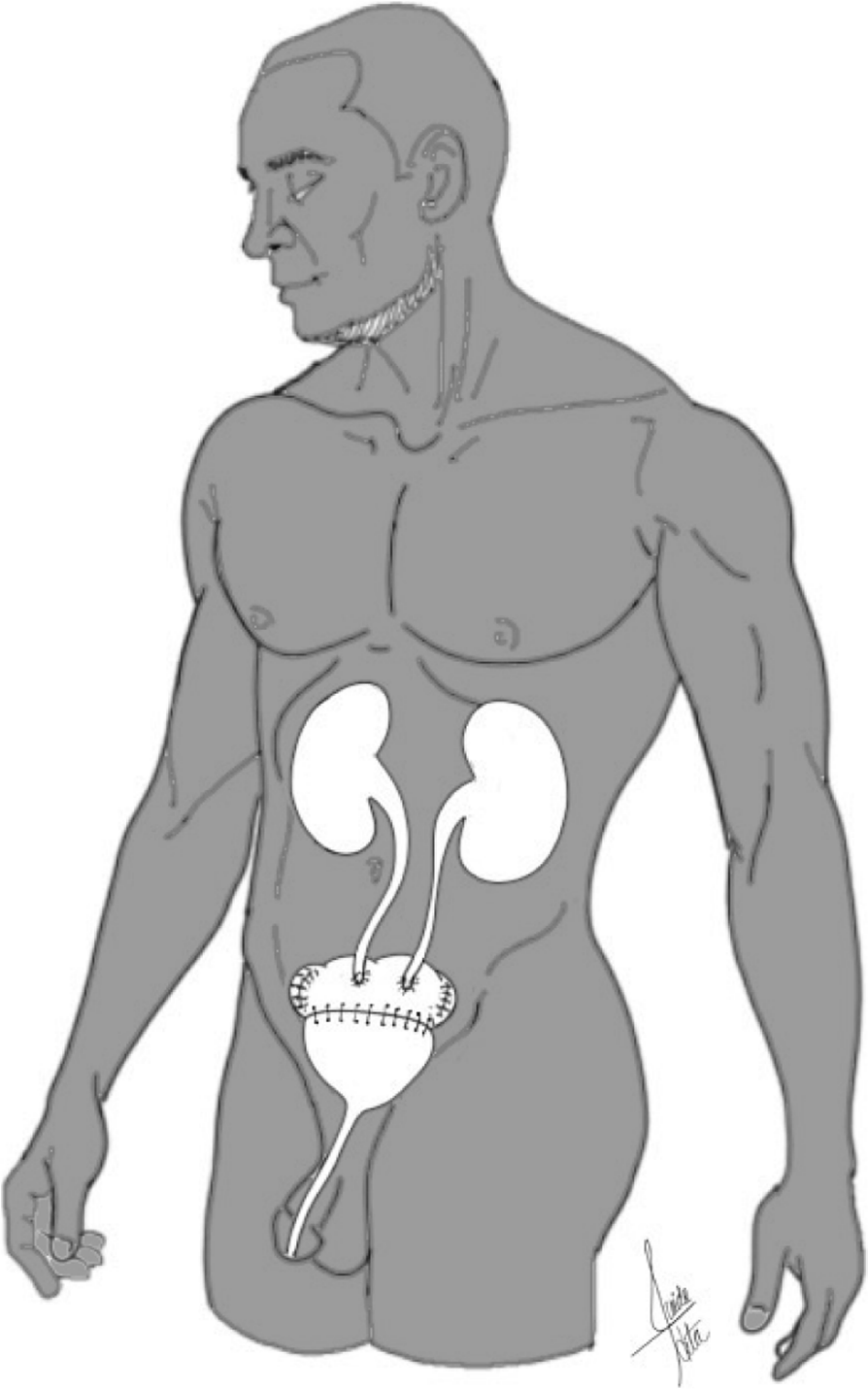
Continent orthotopic diversion. A section of the bowel is used to reconstruct the bladder, which then allows the use of the native urethral sphincter

Urinary stomas are indicated in patients who require urinary diversion, either as a result of radical cystectomy due to malignant conditions (e.g., bladder cancer, urothelial cancer), trauma or benign conditions, such as congenital disorders (e.g., spina bifida) or neuropathic bladder [22]. There are three types of urinary diversions: non-continent cutaneous diversion, continent cutaneous diversion, and continent orthotopic diversion (Figs. 4, 5, and 6), and the first 2 types will have urostomies. Continent diversions require the urinary pouch to be drained 4 to 5 times a day with a thin flexible catheter.

All three types use a bowel segment to create a conduit or pouch or to reconstruct a neobladder. Due to its simple mobilization and extensive mesentery, the ileum is the most frequently utilized bowel segment, but other colon segments, such as the sigmoid colon, also have been used [22].

Despite decades of experience and advances in surgical techniques, medical, and surgical complications are common to all types of urinary diversion. Risk factors for postoperative complications include age, co-morbidities, female sex, obesity, and previous radiation to the abdominal or pelvic region [23–25]. Complications arising within the first 90 postoperative days are considered early complications, whereas those occurring after 90 days are defined as late complications [23].

### Early complications

The incidence of early complications from urostomy is reported to be 20–57% [26]. Up to two thirds of patients experience at least one complication within the first 90 days after the procedure, and approximately 20% experience major complications in the early period [27]. Early complications include gastrointestinal complications, infections, and wound problems that are often related to the surgical procedure [23–28].

#### Gastrointestinal complications

Because the bowel is used for urinary diversion, gastrointestinal complications are very common, occurring in up to 30% of patients during the early postoperative period. Gastrointestinal complications include paralytic ileus, bowel obstruction or anastomotic leakage [25–29]. Patients may present to the emergency department with nausea, vomiting, or fever and signs of sepsis. A full work up in the emergency department is warranted, including imaging and blood work in order to diagnose an obstruction, bowel ischemia, or peritonitis. Placement of a nasogastric tube can be helpful to achieve bowel rest, and intravenous hydration and replacement of electrolytes can replenish losses [29, 30]. In patients with ischemia or peritonitis, antibiotics should be started without delay followed by immediate consultation with the primary team.

#### Infections

Infection is also a common complication in the early postoperative period, occurring in almost 30% of cases [25, 26, 30, 31]. It is important to distinguish between bacterial colonization of the neobladder and symptomatic upper urinary tract infection. Bacterial colonization of the neobladder and asymptomatic bacteriuria have been reported in 78% of patients [24]. In the absence of symptoms, such as fever, chills, and abdominal or flank pain, asymptomatic bacteriuria should not be treated routinely, in order to avoid side effects and the development of antibiotic resistance [24]. However, severe infections can arise from bacterial overgrowth or urinary leakage from the uretero-ileal anastomosis, resulting in wound dehiscence, abscess formation, pyelonephritis, and even sepsis [28, 30]. Any sign or symptom of infection should prompt the emergency physician to undertake a full infectious work up, including bloodwork, blood and urine cultures, and chest and abdominal imaging [25]. Urine should not be tested from the ostomy appliance, as it is not sterile and could produce false positive results; ideally, the urostomy should be catheterized in order to obtain sterile urine. The patient should be resuscitated and started on antibiotics according to local protocols and guidelines within 1 h of presentation and should then be referred to the primary team.

### Late complications

The most frequently observed long-term complications of urostomy include stomal complications, metabolic complications, and mechanical complications including strictures or stenosis around the anastomosis [23, 24, 28, 31].

#### Stoma complications

Stomal complications remain one of the major challenges associated with urinary diversion. They have a huge impact on quality of life and are the most common problem associated with conduit surgery [32, 33]. Urostomy complications include stomal retraction, stenosis or obstruction, herniation, prolapse, and peristomal skin irritation [28, 33]. Most cases will occur within 2 years post-surgery. Hernias are the most common complication in these patients and are caused by fascial defects surrounding the conduit [33]. Contributing factors include obesity, advanced age, and malnutrition [25]. Although parastomal hernias are typically asymptomatic, up to 30% of patients may complain of abdominal discomfort, bulge, or bowel symptoms [33].

Stomal stenosis may result from long-term ischemia, skin irritation from chronic cutaneous exposure to urine, stoma retraction, or fascial narrowing [33]. Clinically patients may complain of difficulties with catheterization and urinary drainage.

In the emergency department, the patient should receive full blood work and imaging tests to exclude obstruction or infection. Early involvement of the stoma nurse and the primary care team are extremely important in order to provide early management of complications and to facilitate coordinated follow-up.

#### Metabolic complications

Because the ileum is responsible for the absorption of vitamins, minerals, and bile salts, urinary diversion can be associated with serious metabolic complications, including electrolyte and metabolic disturbances, nutritional deficiencies, bone disorders, and urolithiasis [23, 34–36]. The severity of those complications depends on the length of the bowel segment used [35, 37].

Since vitamin B12 absorption occurs primarily in the terminal ileum, patients are at risk of vitamin B12 deficiency. It usually takes 3–5 years for the liver’s vitamin B12 stores to be depleted enough to produce symptoms [24, 34]. Depleted patients may present to the emergency department with hematological derangements and neurological deficits, such as peripheral neuropathy, optic atrophy, degeneration of the spinal cord, or dementia [34]. Blood tests, including testing for vitamin B12 levels, can help to confirm the problem. Once vitamin B12 deficiency has been confirmed, lifelong intramuscular injections are required [34].

The use of intestinal segments for urinary diversion is associated with other long-term metabolic consequences. On exposure to urine, the ileum absorbs ammonium and chloride and excretes bicarbonate resulting in hyperchloremic metabolic acidosis in 70% of patients with intestinal urinary diversions [38, 39]. Clinically patients may present to the emergency department complaining of fatigue, lethargy, weakness, or weight loss [37]. Treatment in the emergency department includes correction of the acidosis with sodium bicarbonate or sodium citrate and replacement of the electrolytes and hydration.

In response to the metabolic acidosis, calcium is being released from the bones to buffer the protons. Ileal resection may impair calcium absorption and further reduce bone mineral density, imposing a 21% greater risk of fractures [38]. Currently, no guidelines are available for the management of osteoporosis in this patient population [38].

Patients with urinary diversion are at higher risk for developing kidney stones in response to metabolic changes, chronic infections, urinary stasis, and the presence of foreign bodies, such as sutures or staples [25, 38, 39]. These patients may present with pain, infections, hematuria, obstruction, or difficulties emptying the pouch [25]. Analgesia, hydration, and correction of the electrolytes should be started in the emergency department, followed by a diagnostic work up. The primary care team should be consulted to facilitate definitive treatment [25].

#### Mechanical complications

Mechanical complications include ureteroenteric stenosis and strictures [23, 33]. Ureterointestinal strictures occur in up to 30% of patients and are frequently seen at the site of the anastomosis [35]. They appear within a few months of the procedure up to several years thereafter and are believed to be caused by to ischemia [25]. Clinically patients may present with obstruction, hydronephrosis, flank pain, and infection resulting in pyelonephritis [25, 33]. The patient should receive a full work up, including blood-work, imaging, and referral to the primary team for further management.

## Conclusion

Surgical stoma creation is a common procedure. Stoma-related complications are a common presentation to the emergency department and will continue to affect most patients even years after surgery. Hence, it is vital that emergency physicians are familiar with various types of stoma and the complications and emergencies associated with them, in order to assess and manage them appropriately.

## Data Availability

Data sharing is not applicable to this article as no datasets were generated or analyzed during the current study.

## Abbreviations

BMI: Body mass index
